# Supplementary material for: Succession of transiently active tumor‐initiating cell clones in human pancreatic cancer xenografts
Source: EMBO Mol Med. 2017 May 19;9(7):918–32. doi: 10.15252/emmm.201607354 (PMC5494525; doi:10.15252/emmm.201607354)
Supplement: Supplementary file 1 — Appendix [file EMMM-9-918-s001.pdf]

Appendix

1. Appendix Supplementary Figures and Tables

Appendix Figure S1: Histology of xenografted tumors is unchanged in serial transplantation.

Appendix Figure S2: CD133 positive and negative tumor cells are equally capable to form tumors.

Appendix Table S1: Marker expression of adherent primary pancreatic TIC cultures.

Appendix Table S2: Single nucleotide variants in serially transplanted lentivirally marked PDAC xenografts.

Appendix Table S3: Upper (1-ß) confidence bounds, N_u^-, for the number of cells in a non-detected clone.

Appendix Table S4: Range of clone sizes in T_1 (estimated cell numbers).

Appendix Table S5: Comparative gene expression profiling of serum-free and FBS treated adherent cultures.

Appendix Table S6: Cell surface marker expression does not predict tumor-initiating capacity.

2. Appendix Supplementary Methods

3. Appendix Supplementary Statistical Results

4. Appendix Supplementary References

1. Appendix Supplementary Figures and Tables

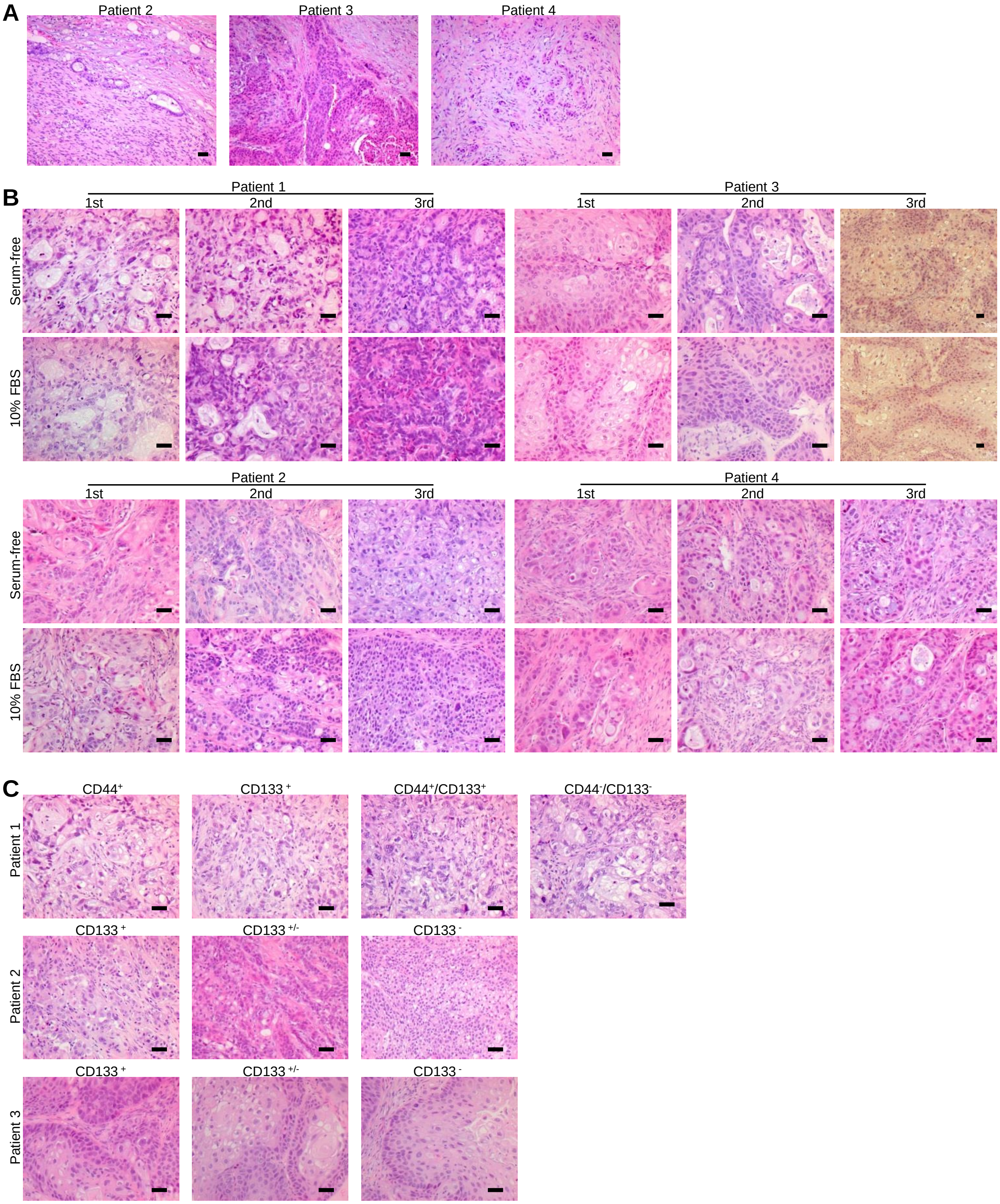

Appendix Figure S1: Histology of xenografted tumors is unchanged in serial transplantation. (A) Original patient tumor histologies. Scale bars = 100µm. (B) No difference in the histology of xenografted tumors after transplantation of pancreatic TIC cultured under serum-free or differentiation conditions (10% FBS). Scale bars = 50µm. 1st, 2nd, 3rd indicate serial mouse generations. Experiment was performed in duplicates. (C) Sorted CD133$^+$ and CD133$^-$ cells formed tumors with similar histology. Scale bars = 50µm.

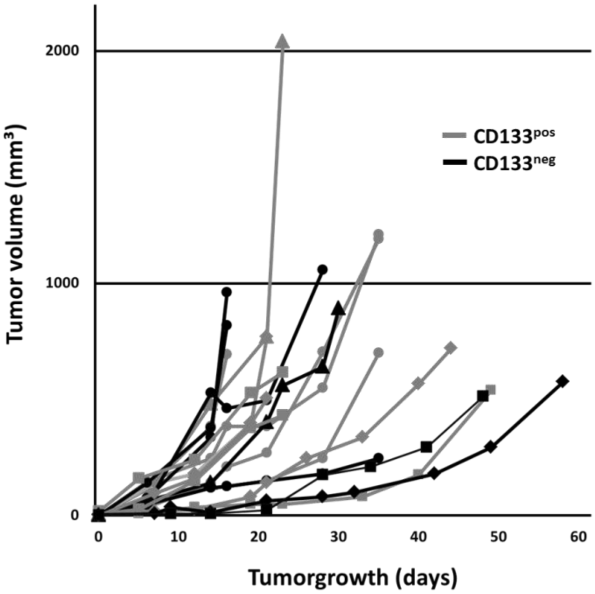

Appendix Figure S2: CD133 positive and negative tumor cells are equally capable to form tumors. Tumor pieces derived from patient P5 (▲), P6 (●), P7(♦) and P8(■) which were not cultivated in vitro before were expanded in NSG mice and sorted according CD133 expression in CD133 positive ($CD133^{pos}$, black lines) and CD133 negative ($CD133^{neg}$, grey lines) fractions. For each patient equal numbers of $CD133^{pos}$, or $CD133^{neg}$, cells were subcutaneously transplanted into NSG mice. Tumor volume was regularly estimated by manual caliper measurements. Mice were sacrificed when tumors reached the maximum tolerable size or when mandated by animal morbidity.

Appendix Table S1: Marker expression of adherent primary pancreatic TIC cultures.

	Patient 1		Patient 2		Patient 3		Patient 4	
**Marker**	**Serum-free**	**10% FBS**	**Serum-free**	**10% FBS**	**Serum-free**	**10% FBS**	**Serum-free**	**10% FBS**
**Flow Cytometry**								
**CD133**	10 - 94%	1 - 55%	11 - 98%	0 - 37%	0.3 - 14%	1 - 45%	12 - 87%	3 - 60%
**CD44**	75 - 100%	96 - 100%	86 - 100%	45 - 100%	87 - 100%	97 - 100%	73 - 100%	70 - 100%
**CD24**	2 - 85%	59 - 96%	0.2 - 22%	0.1 - 10%	60 - 98%	25 - 91%	55 - 94%	55 - 99%
**EpCam**	87 - 100%	96 - 100%	81 - 100%	92 - 100%	90 - 100%	95 - 100%	69 - 100%	95 - 100%
**Indirect Immunofluorescence**								
**KRT7**	+	+	+	+	+	+	+	+
**PTF1a**	+	+	+	+	+	+	-	-
**Amylase**	+	+	n.d.	n.d.	+	+	n.d.	n.d.
**Vimentin**	+/-	+	-	-	-	-	+	+
**Zeb1**	+	+	+	+	+	+	n.d.	n.d.
**E-Cadherin**	+	+	n.d.	n.d.	n.d.	n.d.	n.d.	n.d.
**β-catenin**	+	+	n.d.	n.d.	n.d.	n.d.	n.d.	n.d.
**SOX2**	+/-	+	+	+/-	+	+	-	-
**OCT4**	+	+	+	+	+	+	+	+
**KLF4**	+	+	n.d.	n.d.	n.d.	n.d.	n.d.	n.d.
**Oil droplets**	-	-	-	-	-	+	n.d.	n.d.

Previously described TIC associated cell surface markers were measured by flow cytometry; intracellular markers were analyzed by indirect immunofluorescence staining. Cultures were assigned + when positive cells were detected in at least two independent visual fields of the same sample, +/- when only single positive cells were identified and – if no positive stained cells were detectable. n.d = not determined.

Appendix Table S2: Single nucleotide variants in serially transplanted lentivirally marked PDAC xenografts.

Patient P1					Sequencing Depth					Altered allele frequency				
Chromosome	Position	Reference Allele	Altered Allele	Gene	Patient Tumour	TIC culture	Xenograft 1°	Xenograft 2°	Xenograft 3°	Patient Tumor	TIC culture	Xenograft 1°	Xenograft 2°	Xenograft 3°
1	231061360	C	A	**TTC13**	198	158	141	132	140	0,00	0,00	0,00	0,05	0,02
14	20444084	T	A	**OR4K15**	242	198	172	151	148	0,00	0,00	0,00	0,06	0,03
7	149495370	C	G	**SSPO**	61	56	50	36	44	0,00	0,00	0,00	0,11	0,02
19	519401	C	T	**TPGS1**	48	23	24	21	26	0,00	0,00	0,00	0,14	0,27
19	51628632	G	A	**SIGLEC9**	179	242	208	167	186	0,00	0,01	0,00	0,06	0,08
3	190575666	T	G	**GMNC**	238	256	195	192	174	0,00	0,11	0,09	0,11	0,11
3	164906602	C	T	**SLITRK3**	96	99	126	111	93	0,00	0,13	0,10	0,13	0,13
4	89397167	C	A	**HERC5**	83	93	79	71	77	0,00	0,11	0,22	0,18	0,17
10	77168375	G	A	**ZNF503-AS2**	47	29	38	36	31	0,00	0,31	0,24	0,17	0,35
1	10678409	C	T	**PEX14**	169	128	89	90	101	0,01	0,35	0,35	0,41	0,33

Patient P3					Sequencing Depth					Altered allele frequency				
Chromosome	Position	Reference Allele	Altered Allele	Gene	Healthy Tissue	TIC culture	Xenograft 1°	Xenograft 2°	Xenograft 3°	Healthy Tissue	TIC culture	Xenograft 1°	Xenograft 2°	Xenograft 3°
10	98742139	C	A	**C10orf12**	113	70	43	44	36	0,00	0,00	0,00	0,14	0,17
6	54254685	G	A	**TINAG**	153	119	66	54	115	0,00	0,15	0,02	0,00	0,03
17	40117070	G	A	**TTC25**	83	102	75	58	47	0,00	0,18	0,08	0,12	0,23
1	17087561	C	T	**MST1L**	135	69	69	52	56	0,00	0,14	0,14	0,12	0,13
20	49621352	G	T	**RP5-955M13.3**	85	109	68	89	66	0,00	0,34	0,25	0,40	0,26
1	32149745	G	T	**COL16A1**	78	60	46	42	24	0,00	0,33	0,28	0,17	0,42
1	114483136	C	T	**HIPK1**	205	210	104	103	111	0,00	0,25	0,30	0,13	0,22
16	19488841	G	T	**TMC5**	82	121	64	78	61	0,01	0,49	0,41	0,51	0,43
16	3615019	G	A	**NLRC3**	71	78	63	50	43	0,00	0,40	0,43	0,46	0,47
15	81591795	G	A	**IL16**	488	683	399	340	310	0,00	0,46	0,46	0,33	0,39
1	50941270	G	A	**FAF1**	85	72	41	43	38	0,00	0,56	0,46	0,49	0,39
16	28133033	T	C	**XPO6**	114	125	66	72	80	0,00	0,43	0,53	0,39	0,44
5	140553101	C	T	**PCDHB7**	299	247	185	146	138	0,01	0,52	0,55	0,48	0,51
11	68458451	C	T	**GAL**	307	335	260	207	166	0,01	0,56	0,57	0,60	0,58
17	44248455	G	C	**KANSL1**	120	169	83	65	66	0,00	0,66	0,58	0,65	0,65
19	50511077	C	T	**VRK3**	51	61	31	29	23	0,00	0,46	0,58	0,59	0,48
2	11798685	G	A	**NTSR2**	148	170	111	103	83	0,00	0,73	0,64	0,57	0,83
11	18309148	T	C	**HPS5**	148	196	101	94	76	0,01	0,59	0,64	0,63	0,63
11	123814014	A	T	**OR6T1**	169	158	83	91	64	0,00	0,53	0,65	0,60	0,59
17	35634884	C	T	**ACACA**	87	117	79	61	49	0,01	0,62	0,66	0,75	0,67
1	155764861	T	A	**GON4L**	107	60	66	53	51	0,00	0,72	0,71	0,79	0,82
9	139272123	C	T	**SNAPC4**	107	88	55	54	46	0,01	0,74	0,82	0,69	0,89
1	186008998	C	A	**HMCN1**	89	94	73	60	53	0,01	0,83	0,85	0,92	0,75
9	21971120	G	A	**CDKN2A**	39	34	20	17	13	0,00	0,79	0,90	0,88	0,77
4	164415698	C	G	**TMA16**	147	90	56	46	41	0,00	0,96	0,93	1,00	1,00
6	30955174	T	C	**MUC21**	145	112	73	69	70	0,01	0,89	0,96	1,00	0,99
15	72186067	C	G	**MYO9A**	132	167	87	103	73	0,00	0,99	1,00	1,00	0,99
12	25398284	C	T	**KRAS**	218	198	110	94	103	0,00	0,97	1,00	1,00	0,99
X	51640014	C	G	**MAGED1**	242	159	87	115	69	0,00	1,00	1,00	1,00	1,00
10	129242520	A	T	**DOCK1**	241	123	71	88	55	0,00	0,98	1,00	1,00	1,00
15	75144528	C	A	**SCAMP2**	60	96	55	45	34	0,02	1,00	1,00	1,00	1,00
15	80988233	C	A	**ABHD17C**	69	107	44	45	42	0,01	1,00	1,00	1,00	1,00
17	7578257	C	A	**TP53**	113	82	64	53	37	0,01	1,00	1,00	1,00	1,00
18	45395781	G	C	**SMAD2**	111	64	67	33	35	0,02	1,00	1,00	1,00	1,00
X	130407781	C	T	**IGSF1**	51	44	20	23	20	0,00	1,00	1,00	1,00	1,00

Exome sequencing of primary culture and subsequent xenograft generations (1°-3°) as well as corresponding healthy tissue control (P3) or original patient tumor tissue (P1) demonstrated a very limited number of newly acquired mutations during serial transplantation. Orange colored fields indicate proportion of altered allele frequencies. Grey colored gene fields mark genes with novel mutations acquired during serial transplantation. Xenograft 1° - 3°= Xenograft generation 1 - 3.

Appendix Table S3: Upper (1-ß) confidence bounds, N_u^-, for the number of cells in a non-detected clone[(*)]

	N_u^-							
	T1		T2a		T2b		T2c	
Experiment	β= 1%	0.01‰	1%	0.01‰	1%	0.01‰	1%	.01‰
P1-1	149	344	191	440	167	385	134	309
P2-1	205	474	243	561	203	468		
P3-1	262	604	273	629	114	262		
P3-2	50	115	100	232	93	215		
P3-3	67	154	74	172	138	319		

[(*)] test-based, Clopper-Pearson type, two-sided. The sampling fractions, i.e. the proportions s of cells in the tumors whose DNA was analyzed for clonality, in the 5 experiments and used in the calculation were: T_1: s=3.48%, 2.54%, 2.0%, 10%, 7.6%; T_{2a}: s=2.73%, 2.15%, 1.92%, 5.12%, 6.85%; $T2_b$: s=3.12%, 2.57%, 4.54%, 5.50%, 3.75%; T_{2c}: s=3.87% (Exp. P1-1)

Appendix Table S4: Range of clone sizes in T_1 (estimated cell numbers).

	Numbers of cells per clone		
Experiment	min (obs.)	max (obs.)	min (present, but non-obs.)[(*)]
P1-1	4,401	509,095	≤ 11
P2-1	10,566	1,013,328	≤ 20
P3-1	75,918	4,357,437	≤ 20
P3-2	20,885	1,946,043	≤ 2
P3-3	378	94,871	≤ 11

[(*)] upper bounds based on confidence bounds shown in Appendix Table S3 (β=0.01‰). Since the minimum sizes multiplied with the number of clones must not exceed the values in Appendix Table S3 the results were rounded to the next lower integer.

Appendix Table S5: Comparative gene expression profiling of serum-free and FBS treated adherent cultures.

	P1						P2					
Stem Cell Associated Markers	3P, serum-free	3P, 10% FBS	fold change	8P, serum-free	8P, 10% FBS	fold change	3P, serum-free	3P, 10% FBS	fold change	8P, serum-free	8P, 10% FBS	fold change
SOX2			-0.46			0.68			-1.04			-0.57
OCT4			0.08			-0.09			0.05			0.09
c-Myc			-0.74			-0.62			-1.01			-0.05
KLF4			-0.61			-1.00			-1.09			0.72
NOTCH1			-1.04			1.00			-1.02			-1.68
ALDH1A1			-1.93			-1.60			-0.63			-2.07
ALDH1A2			-0.23			0.43			-0.40			-0.05
ALDH1A3			3.92			4.88			0.78			2.28
PROM1			-0.98			-1.36			-2.74			-1.96
CD44			-0.06			0.02			-0.26			-0.43
EpCam			-0.27			-1.04			-0.85			-0.32
CD24			0.27			1.77			0.87			-0.51
CXCR4			-0.15			-0.08			-0.13			-0.03
Panreatic Progenitor Associated Markers												
SOX9			-0.91			-0.80			-0.95			-1.27
PDX1			-0.32			-0.22			-0.03			0.05
HES1			-0.87			-0.73			-1.21			1.54
												0.00
Ductal differentiation markers												0.00
KRT7			0.41			0.39			2.81			2.94
KRT19			-0.60			-1.09			0.37			-0.02
CEACAM1			-0.86			-0.37			0.24			0.82
CEACAM5			-2.96			-5.09			2.38			1.44

	P3						P4					
Stem Cell Associated Markers	3P, serum-free	3P, 10% FBS	fold change	8P, serum-free	8P, 10% FBS	fold change	3P, serum-free	3P, 10% FBS	fold change	8P, serum-free	8P, 10% FBS	fold change
SOX2			-1.06			-1.64			0.01			0.45
OCT4			-0.02			-0.14			0.13			-0.18
c-Myc			-0.69			-0.18			-0.06			2.80
KLF4			-0.34			-1.35			-1.03			0.44
NOTCH1			-0.64			-0.54			0.24			-0.11
ALDH1A1			-0.70			-0.31			-0.51			-0.02
ALDH1A2			0.01			0.06			-0.03			0.09
ALDH1A3			1.32			2.63			2.67			0.54
PROM1			0.68			1.52			-1.48			0.27
CD44			-0.20			-0.02			-0.69			0.31
EpCam			0.21			-0.26			0.28			0.46
CD24			-0.41			-0.08			0.32			-0.13
CXCR4			0.05			-0.48			-0.03			0.05
Panreatic Progenitor Associated Markers												
SOX9			-1.77			0.05			0.20			0.82
PDX1			-0.19			0.52			-0.22			-0.03
HES1			-0.91			-1.92			0.98			0.31
Ductal differentiation markers												
KRT7			1.22			1.90			1.62			0.29
KRT19			-0.42			-0.51			0.82			0.00
CEACAM1			-2.32			1.16			1.10			0.66
CEACAM5			-3.48			-0.33			1.14			0.63

6 7 8 9 10 11 12 13 14

Absolute expression

Fold change of gene expression in serum treated cells (10% FBS) compared to control cultures (serum-free). Color code indicates levels of absolute expression values. An at least 2-fold increase is highlighted in light red and at least 2-fold decreased expression is highlighted in light blue fold change numbers. Analysis was performed in culture passage 3 (3P) and 8 (8P). P1 - 4: patient number 1 - 4.

Appendix Table S6: Cell surface marker expression does not predict tumor-initiating capacity.

Patient 1

Fraction	Sorted from	Tumour (g)	CD44 in vivo (%)	CD133 in vivo (%)
CD44$^-$/ CD133$^-$	Serum-free	1.9	6.2	42.4
CD44$^+$/ CD133$^-$	Serum-free	0.9	6.5	36.1
CD44$^-$/ CD133$^+$	Serum-free	1.2	5.7	39.4
CD44$^+$/ CD133$^+$	Serum-free	1.6	4.2	35.1

Patient 2

Fraction	Sorted from	Tumour 1 (g)	CD133 in vivo (%)	Tumour 2 (g)	CD133 in vivo (%)
CD133$^-$	Serum-free	0.8	31.9	0.6	n.d.
CD133$^{+/-}$	Serum-free	0.45	20.8	0	
CD133$^+$	Serum-free	0.15	21.7	0	

Fraction	Sorted from	Tumour 1 (g)	CD133 in vivo (%)	Tumour 2 (g)	CD133 in vivo (%)	Tumour 3 (g)	CD133 in vivo (%)
CD133$^-$	10% FBS	0.4	22.8	0.6	21.9	0.1	n.d.

Patient 3

Fraction	Sorted from	Tumour 1 (g)	CD133 in vivo (%)	Tumour 2 (g)	CD133 in vivo (%)
CD133$^-$	10% FBS	1.1	0.2	0.7	0.7
CD133$^{+/-}$	10% FBS	0.35	0.2	0.3	0.4
CD133$^+$	10% FBS	0.4	0.2	0.6	0.5
CD133^{--}	10% FBS	0.5	0.2	n.d.	

Sorted CD133$^+$ and CD133$^-$ cell fractions are equally able to form tumors in immune-deficient mice. Tumors grown from highly purified CD133$^-$ and CD133$^+$ cells contained the same proportion of cells expressing CD133 (columns: CD133 in vivo). For patients 2 and 3 tumors were transplanted in duplicates or triplicates (columns: Tumor 1/2/3). Tumor weights are indicated in gram (g). Beside CD133 positive enriched (CD133$^+$) intermediate fractions (CD133$^{+/-}$) were sorted. CD133 negative cell fractions were sorted once (CD133$^-$) or twice (CD133^{--}) for enrichment.

2. Appendix Supplementary Methods

1. Statistic methods

1. General Remarks

The statistical analysis focused exclusively on transduced cells; i.e., in what follows the terms “tumor”, “sample”, and “clone” solely refer to transduced cells. The following three null hypotheses were tested:

$\mathbf{H_{0,P1}}$: Proliferation rates of all clones in a tumor are identical;

$\mathbf{H_{0,P2}}$: Proliferation rates of all clones in the primary or secondary mice, while possibly heterogeneous, are identical to the proliferation rates of the same clones in the next mouse generation (“constant growth rates”).

$\mathbf{H_{0,S}}$: The seeding efficiencies of all clones in a tumor are identical.

In addition to standard methods, such as Fisher’s exact test for equality of proportions and Clopper-Pearson confidence intervals for proportions, we employed techniques that were tailored to the special situation encountered here, characterized, e.g., by complex serial sampling, the absence of longitudinal observations on clone growth, unknown number of clones present in the tumors, and the need to simultaneously account for observations gathered from different mouse generations:

1.1. Confidence interval p-values. This is an important tool for worst-case analyses. The concept was developed by Berger and Boos (Berger & Boos, 1994), and in some way constitutes an improvement over the old idea of supremum p-values originally formulated by Barnard (Barnard, 1947), which stated that when using a statistical test involving a nuisance parameter Θ, a valid p-value can be obtained by maximizing the conditional p-values $p(\Theta)$ over the parameter space of Θ. Berger and Boos showed that restricting the maximization of the conditional p-values to a $1-\beta$ confidence C_β set for Θ (obtained when the null hypothesis is true), and adding ß to this maximum, also results in a valid p-value (the “confidence interval p-value” see also (Berger et al, 2010; Lin & Yang, 2009).

1.2. Test-based confidence intervals for the parameter n of a binomial distribution B(n,p). We used these intervals to determine upper confidence limits for the number of clones in a given tumor T. The procedure was as follows: Let H_0 be the null hypothesis that all cells of T have the same probability *s* of being contained in the sample S of cells examined for the existence of clones, and assume H_0 to hold true. Then, for a given total number n_0 of tumor cells not contained in S, the probability that none of the clones corresponding to these cells is detected in S is equal to the probability that none out of any given set of n_0 tumor cells is contained in S_1.

Given s, the upper bound of a two-sided test-based level $(1-\beta)$ confidence interval for the number of clones not detected in S can thus be determined using a binomially distributed variable $X \sim B(n,s)$, X being the number of cells (from a sample of n cells) which are detected in S, and is calculated as the highest number $n=n_u$ of cells such $P(X=0) \geq \beta/2$. It is easily seen that $n_u = \log(\beta/2)/\log(1-s)$. The upper two-sided $(1-\beta)$ confidence limit for the number of clones in T is then calculated as n_u plus the number of clones contained (i.e., “detected”) in S.

1.3. Supremum p-values over possible constellations of unobservable count data. This is a special case of supremum p-values, the missing numbers being regarded as nuisance parameters.

1.4. Modelling of the cell growth process. We modelled the cell growth process by means of a birth process (a Yule process, i.e. a Poisson process for the number of divisions a single cell) with an identical growth parameter for all cells of a clone. In view of the short time interval between transplantation and harvesting of the tumors and the scarcity of data available for estimating unknown parameters, cell death was not incorporated in the model. Within this model, null hypotheses regarding proliferation rates can then be expressed in terms of the growth parameters λ, i.e. the rate of the Poisson process. E.g., the null hypothesis of homogeneity of proliferation rates states that the

parameters λ_i of the processes are the same for each clone i. Clone size distributions were obtained by using known formulas for the Yule process (Bharucha-Reid, 1967) (p.403), which imply that cell numbers after a fixed time follow a negative binomial distribution NB(r,p), with r= number of cells at t=0 and p=exp(-λt) .

Two approximations were used throughout the analysis:

A) the number of transduced cells (among all cells of a tumor or a sample hereof) was set to be equal to the expected value calculated as the total cell number in the tumor or sample multiplied with the proportion of transduced cells, the latter having been determined in a separate analysis based on about 10,000 cells;

B) in order to describe sampling from a clone the binominal distribution was used when, in fact, the sampling depended on the total complex clone structure of a tumor, i.e. followed a multivariate hypergeometric distribution. The approximation was justified by the low probabilities of selection (ranging from 1.92% to 10% in primary or secondary mice). Adjustment for multiplicity was restricted to situations where several results (e.g., inferences for each clone in a tumor) were used in the same analysis, i.e. for testing the same hypothesis. No adjustment for multiple testing was done regarding the multiplicity of different hypotheses or experiments conducted.

2. Details of the analysis

Notation: Let (for i=1,…,3) M_i designate primary, secondary, and tertiary mice (respectively), T_i the tumors of these mice, S_i the samples taken from these tumors and analysed for clonality, CS_i the clones detected in S_i, CNS_i the clones present in T_i but not detected in S_i, and CNS_i* the subset of the latter which were detected in samples of later mouse generations. Letters a,b,c (e.g. T_{2a},T_{2b}) will be used to differentiate between tumors and samples taken from several secondary mice (and analogously for tertiary mice). Let k(…) and n(…) denote the numbers clones and cells (respectively) in a sample of cells or a set of clones. If not stated differently, cell numbers will refer to tumors, not samples taken from tumors; thus, e.g. n(CNS_1) designates the number of cells in T_1 contained in clones that were not detected in the sample S_1.

2.1. Heterogeneity of proliferation rates (null hypothesis: H_{0_P1})

In order to ascertain the heterogeneity of proliferation rates in T_1 we first determined an upper confidence bound, λ_u, for the parameter of the process under the assumption of H_{0_P1} that all clones in T_1 had the same growth parameter. The upper bound was test-based. The idea underlying the procedure was that once the common growth parameter λ (and thus the expected clone size at time t_s of sampling) exceeded a certain level, this would no longer be statistically compatible with the rather high number of clones (namely, at least k(CNS_1*) many) not present in the sample.

λ_u was calculated using exact probabilities p_0 that a particular clone is undetected in S_1, which

is given by

$$p_0(\lambda, s) = \sum_{i=0}^{\infty} P(X(t)=i)P(Y_i = 0)$$

where X(t) is size of this clone in T_1 after time t and starting with a single cell at t=0 (i.e., X(t) follows a negative binomial distribution NB(1,exp(-λt))), and Y_i describes the sampling from this clone. Hence, Y_i is binomially distributed as Y_i~B(i,s), s being the proportion of cells analysed for clonality (the “sampling fraction”), which is the ratio of the cell numbers in S_1 to those in T_1. Thus, a valid test-based level (1-β) confidence bound λ_u is given by the lowest value (of all discrete values examined) such that high observed ratio of undetected clones, namely,

$$R = \frac{k(CNS_1^*)}{k(CNS_1^*) + k(CS_1)}$$

is statistically "incompatible" with the probability $p_0(\lambda,s)$, meaning that $P(Z \geq CNS^*_1) < \beta/2$, where $Z \sim B(k(CNS_1^*) + k(CS_1), p_0(\lambda,s))$.

Of note, the ratio R ratio is not equal to the exact proportion of clones not present in the S_1, because neither the nominator nor the denominator includes unobserved clones contained in $CNS_1 \backslash CNS_1^*$. However, each of these clones would increase both the nominator and denominator by 1 and thus increase the proportion. Thus, a statistical test taken this modification into account would have an even lower p-value.

The time interval from transplantation (t=0) to sampling (t_s) was rescaled to one time unit. The growth parameter λ then is the mean number of cell divisions until t_s.

The value of λ_u, calculated at the $1\text{-}10^{-5}$ confidence level, was then investigated to determine whether, conversely, it was statistically compatible with the very high observed clone sizes in CS_1. To obtain p-values we only considered the maximum observed clone size, $c_{max,}$. While the true number $k(S_1)$ was unknown, for any assumed number $k{:=}k(S_1)$ and given growth rate the probability distribution function $F_\lambda(x)$ of the maximum clone size could be calculated using elementary results from extreme-value theory, viz. $F_\lambda(x) = G_\lambda(x)^k$, where $G_\lambda(x)$ is the distribution function of the cell number of a clone after time t_s. The value of k used for the analysis was the sum of clones observed plus the upper ($1\text{-}10^{-5}$) confidence bounds for the number of unobserved clones. Following the confidence interval p-value principle, 1-the confidence levels used for the nuisance parameters, i.e. $2*10^{-5}$, was then added.

2.2. Changes in proliferation rates (null hypothesis: H_{0_P2})

The analysis aimed to show that there was at least one clone in T_1 whose proliferation rate increased in T_2. We based our analysis on the clone in CNS_1^* that was largest in CS_2, along with a Bonferroni adjustment for the multiple testing implicitly involved in this particular choice.

The analysis accounted for the fact that, in contrast to T_1, clones in T_2 originated from more than one transplanted cell. The number of cells transplanted into the secondary mice and starting the growth process was itself a random variable, namely the result of a sampling process in the primary tumor. However, cell growth in T_1 and T_2 could be modelled as independent processes, due to the known property of Poisson processes of being memoryless.

The null hypotheses tested was a joint hypothesis, stating that sampling from tumors was random, and the proliferation rates of each clone in T_1 were unchanged in T_2. We chose a statistical test based on the pair (X_1,X_2) of the observed sizes X_i of the same clone in S_1 and S_2, respectively. The rejection region was such that it simultaneously reflected the fact that (under H_0) at least one of the two clone sizes was too extreme to be compatible with any assumed proliferation rate λ (resp.). In view of the independence of the growth processes in T_1 and T_2, the p-value was calculated as a product of probabilities namely p_1*p_2, where $p_1=P(X_1 \leq c_1 | H_0)$ and $p_2=P(X_2 \geq c_2 | H_0)$. Both p_1 and p_2 were calculated form identical clone size distributions, i.e. assuming identical growth rates and growth times (see the remarks below). The rejection region was thus defined by one-sided probabilities taken from the cumulative distribution of X_i under H_0. The particular definition of the test statistic implies that only *increases* in clone sizes from T_1 to T_2 were considered when calculating p-values. The single p-values $p_1=P(X_1 \leq c_1)$ and $p_2=P(X_1 \geq c_2)$ were then determined using the clone size distribution in S_1 and S_2. From the definition of CNS_1^* it follows that $c_1=0$, while c_2 was obtained by multiplying $n(S_2)$ with the observed proportion of the clone in S_2. By construction p_1 will be small for high growth rates, while p_2 will be small for low growth rates of a clone. Therefore, the product p_1p_2 as a function of the growth rate λ has a maximum. This maximal value was determined and used as an upper bound for the p-value. The result was then adjusted for multiplicity, using upper 99% confidence bounds for the number of clones in CNS_1.

The analysis was restricted to P1-1, P2-1, P3-1, and P3-2, where the time interval from transplantation to purification in secondary mice was at most as long as in primary mice. Note that the shorter this interval the smaller the clones. This implies that the probability p_2 calculated under the assumption of identical clone size distributions in T_1 and T_2 is an upper bound for the true value of p_2 if proliferation time in T_2 is shorter than in T_1.

2.3. Heterogeneity of the seeding efficiency (null hypothesis: H_{0_S})

We defined seeding efficiency at the clone level. The following definition was used: Seeding efficiency (SE) = probability that a randomly chosen cell of a clone that is transplanted into a mouse proliferates or survives until the time t_s when the tumor is examined for clonality.

All inferences regarding the SE of a clone had to be deduced from two data points, namely the estimated clone sizes at times t_0 (transplantation) and t_s (cell sampling from the tumor).

We focused the analysis on T_2, and, more specifically, on the first secondary mouse M_{2a}. The analysis was aimed at showing that a lower bound for the SE (in T_{2a}) of cells in clones contained CNS_1 was significantly higher than an upper bound for the SE of at least one clone in CS_1 (while adjusting for multiplicity of testing).

A lower bound for the SE of cells in CNS_1 was obtained by observing that the nominator n_A of SE, i.e., the number of cells in (clones of) CNS_1 successfully transplanted into M_{2a} was at least as large as the number clones in CNS_1 observed either in M_{2a} or its corresponding tertiary mice. To obtain an upper bound d_A for the denominator the number of cells in CNS_1 was replaced with their upper 99.9% confidence bound. Thus, at the 99.9% confidence level for the denominator the SE of cells in CNS_1 was at least n_A/d_A. The clone selected for comparison was the largest clone C in $CS_1 \cap CNS_2$. A lower 99.9% confidence bound, p, for its clone proportion of C in T_1 was calculated based on the observed proportion of C in S_1. Multiplying p with the number of cells of T_1 transplanted into the secondary mouse yielded a lower 99.9% confidence bound, d_B, for the number of cells in C transplanted into the secondary mouse. This number served as the denominator for calculating an upper bound for the SE of C. Since C was undetected in T_2, an upper 99.9% confidence bound for the size of C in T_2 was obtained as described above. This yielded an upper bound for the number of cells in C successfully seeded and was used as the nominator n_B for the SE of C in M_{2a}. The ratios d_A/n_A and d_B/n_B were then compared using an exact test, and the resulting p-value was adjusted for multiplicity involved in the particular selection of C (a Bonferroni adjustment with the total number of clones in CS_1 being the adjustment factor).

3. Appendix Supplementary Statistical Results

Upper bounds for the number of clones in a tumor

Upper confidence bounds, N_u^-, for the number of cells, N^-, in clones present, but not detected in tumors T_1 and T_{2a}, T_{2b}, T_{2c} of primary and secondary mice, respectively are given in Supplemental statistical results Appendix Table S3.

At the same time, these numbers may be viewed as upper bounds for the total number of clones contained in CNS (assuming the worst case that each of these clones consist of a single cell). In contrast to CNS_i, the values CNS_i* (i=1,…,3) were observable. In case of P1-1, e.g., CNS_1* contained 30 clones. The numbers given in Appendix Table S3 impose upper limits to the (mean) size of clones in CNS_i*, which are obtained by dividing N_u^- by the number of clones in CNS_i*, yielding, e.g., at level 1-β=99%, a mean size of about 5 in case of the primary mouse of P1-1. In other words, clones that were definitely present, but non-detected were few and mostly extremely small, a fact that was evident even without a numerical analysis.

Clone sizes within a tumor were quite heterogeneous. Appendix Table S4 shows the estimated sizes (cell numbers) of the largest and smallest clones detected in S_1 as well as an upper bound for the smallest clone in CNS_1. For clones observed in S_1, the numbers were simply obtained by multiplying the clone size proportions in S_1 with the number of cells in T_1. For clones in CNS_1, the upper confidence bounds from Appendix Table S3 were used and divided by the minimum number of clones contained in CNS_1, viz. the number of clones in CNS_1*.

Heterogeneity of proliferation rates

Based on the minimum number of unobserved clones and assuming the null hypothesis of homogeneous proliferation rates λ across the clones of T_1, a value of λ_u=5 was obtained as an upper bound for the common rate at the (1-10^{-5}) confidence level for each experiment. However, using the extreme-value distribution with the total number of clones being limited by the observed clones plus the values in Supplemental statistical results Appendix Table 1 (again at the (1-10^{-5}) confidence level), λ=5 was not compatible with the size of the largest clone T_1 ($p<10^{-8}$ based on the negative binomial distribution). Adding 1-confidence levels of the nuisance parameters (twice 10^{-5}), the result remained highly significant (confidence interval p-value <10^{-4}) for each experiment.

Changes in proliferation rates

The statistical analysis focused on those clones in $CNS_1^* \cap CS_2$ which were largest in CS_2. One may note that the change in growth rates from T_1 to T_2 would be evident for these clones if sampling of cells from the tumor were the only source of random variation for their observed size (which would be the case if clone growth were adequately described by a non-stochastic differential equation). The reason is that clones in CNS_1* were necessarily very small (see Appendix Table S3,4), while those in CS_2 were large. Under the null hypothesis that growth of every clone remained unchanged from T1 to T2, the nominal p-value, calculated as a confidence interval p-value (with the true unknown clone size in T_1 (and thus also in T_2) being the nuisance parameter) was <10^{-6} in each experiment and remained significant at the 0.001 level after adjustment for the multiplicity of tests.

Under our model of clone growth as a Poisson process, which implies a high variability of true clone sizes - even for clones with identical growth rates -, the analysis was more complex. Carrying out the procedure as described, the upper bounds for the nominal p-values obtained for P1-1, P2-1, P3-1, P3-2 were 0.00033, 0.000056, 0.000034, and $3.8 \cdot 10^{-6}$, respectively. Except for P1-1 they remained significant at the α=0.05 level after adjusting for multiplicity and the use of confidence interval p-values.

Analysis of seeding efficiency

The analysis of the heterogeneity of clones with respect to seeding efficiency focused on seeding efficiency in M_{2a}. A statistical comparison between a lower bound for the seeding efficiency (in M_{2a}) of clones in CNS_1 versus an upper bound for the largest clone (largest with respect to T_1) in $CS_1 \cap CNS_2$ was performed for the first 4 experiments. It yielded nominal p-values of $p=0.0001$ in case of P1-1, and $p<10^{-6}$ in case P2-1, P3-1, and P3-2, and thus remained significant ($p< 0.01$) after adjusting for the use of confidence interval p-values and multiplicity of testing. This demonstrates that transplanted clones were heterogeneous with respect to seeding efficiency in secondary mice.

4. Appendix Supplementary References

Barnard GA (1947) Significance Tests for 2x2 Tables. *Biometrika* 34**:** 123-138

Berger MF, Levin JZ, Vijayendran K, Sivachenko A, Adiconis X, Maguire J, Johnson LA, Robinson J, Verhaak RG, Sougnez C, Onofrio RC, Ziaugra L, Cibulskis K, Laine E, Barretina J, Winckler W, Fisher DE, Getz G, Meyerson M, Jaffe DB et al (2010) Integrative analysis of the melanoma transcriptome. *Genome Res* 20**:** 413-427

Berger RL, Boos DD (1994) P-Values Maximized over a Confidence Set for the Nuisance Parameter. *JASA* 89**:** 1012-1016

Bharucha-Reid AT (1967) Review: An Introduction To Probability Theory And Its Applications. By William Feller. *Am Math Monthly* 74: 460

Lin CY, Yang MC (2009) Improved p-Value Tests for Comparing Two Independent Binomial Proportions. *Commun Stat Simul Comput* 38**:** 78-91
