# Supplementary material for: Succession of transiently active tumor‐initiating cell clones in human pancreatic cancer xenografts
Source: EMBO Mol Med. 2017 May 19;9(7):918–32. doi: 10.15252/emmm.201607354 (PMC5494525; doi:10.15252/emmm.201607354)
Supplement: Supplementary file 2 — Expanded View Figures PDF [file EMMM-9-918-s002.pdf]

Expanded View Figures

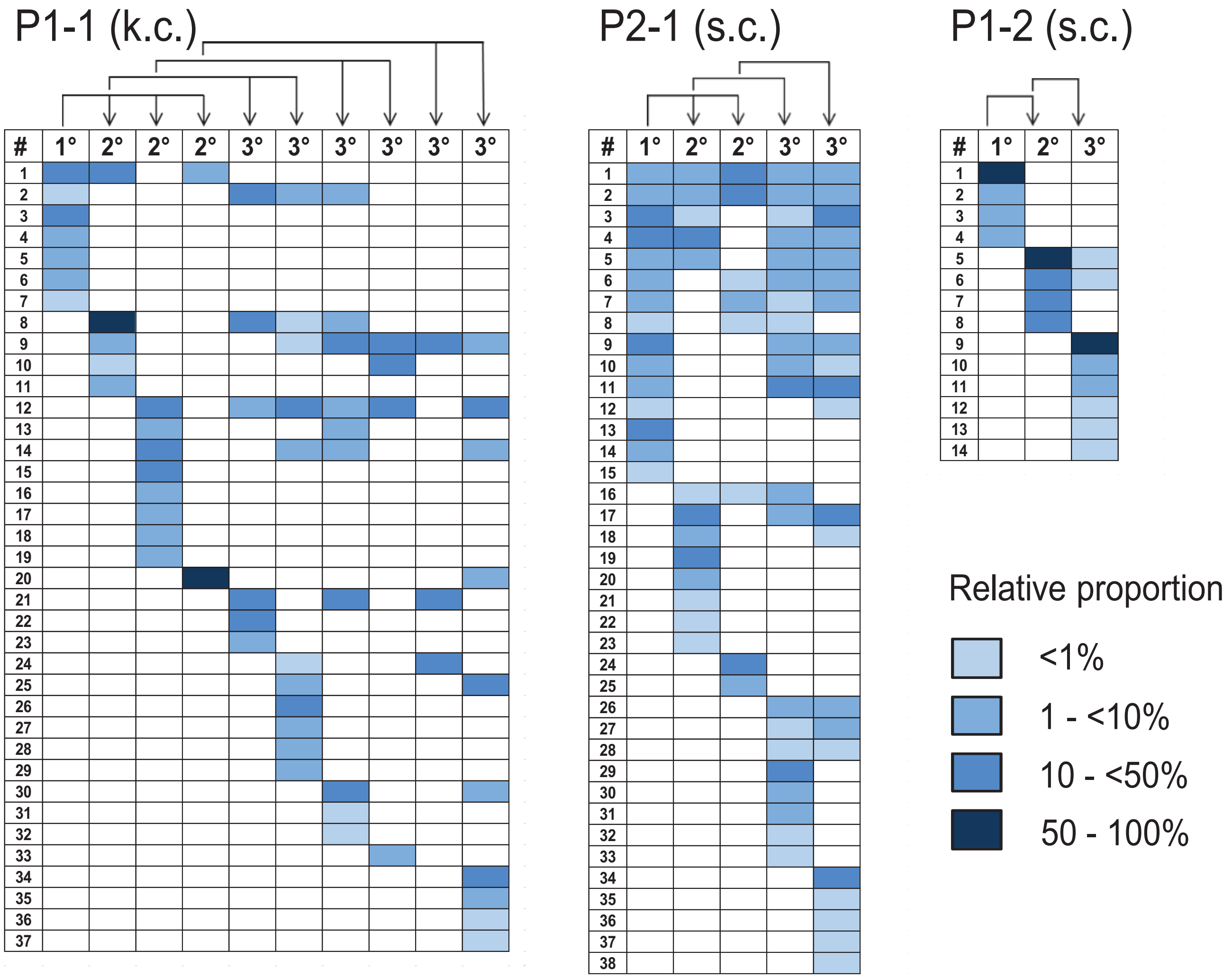

Figure EV1. Long-term tumor growth is maintained by clonal succession of pancreatic TICs.
High-throughput sequencing of lentiviral insertion sites (IS) shows that distinct sets of marked clones formed serial tumors derived from the same individual primary xenograft. Columns indicate distinct mouse generations (1°/2°/3°). Rows indicate distinct lentiviral integration sites. Arrows indicate serial transplantation steps; PX-Y: X = patient number and Y = experiment number.

Figure EV2. Proliferation of TIC cultures *in vitro* is driven by clonal succession.

A Patient-derived adherent TIC cultures were lentivirally marked and passaged 8 times *in vitro*.
B Lentivirally marked TIC cultures were transplanted directly into NSG mice. After tumor purification, cells were taken into culture for several passages (*ex vivo*). IS analysis was done by LAM-PCR.

Data information: In serial passaging, distinct sets of tumor cells drive proliferation of *in vitro* (A) and *ex vivo*-cultured lentivirally marked xenografts (B). Blue fields indicate the relative contribution of individual clones as depicted in the color legend. Columns indicate distinct passages (1–8°). Rows indicate distinct lentiviral integration sites. Arrows indicate passaging steps; PX-Y: X = patient number and Y = experiment number.

 © 2017 The Authors

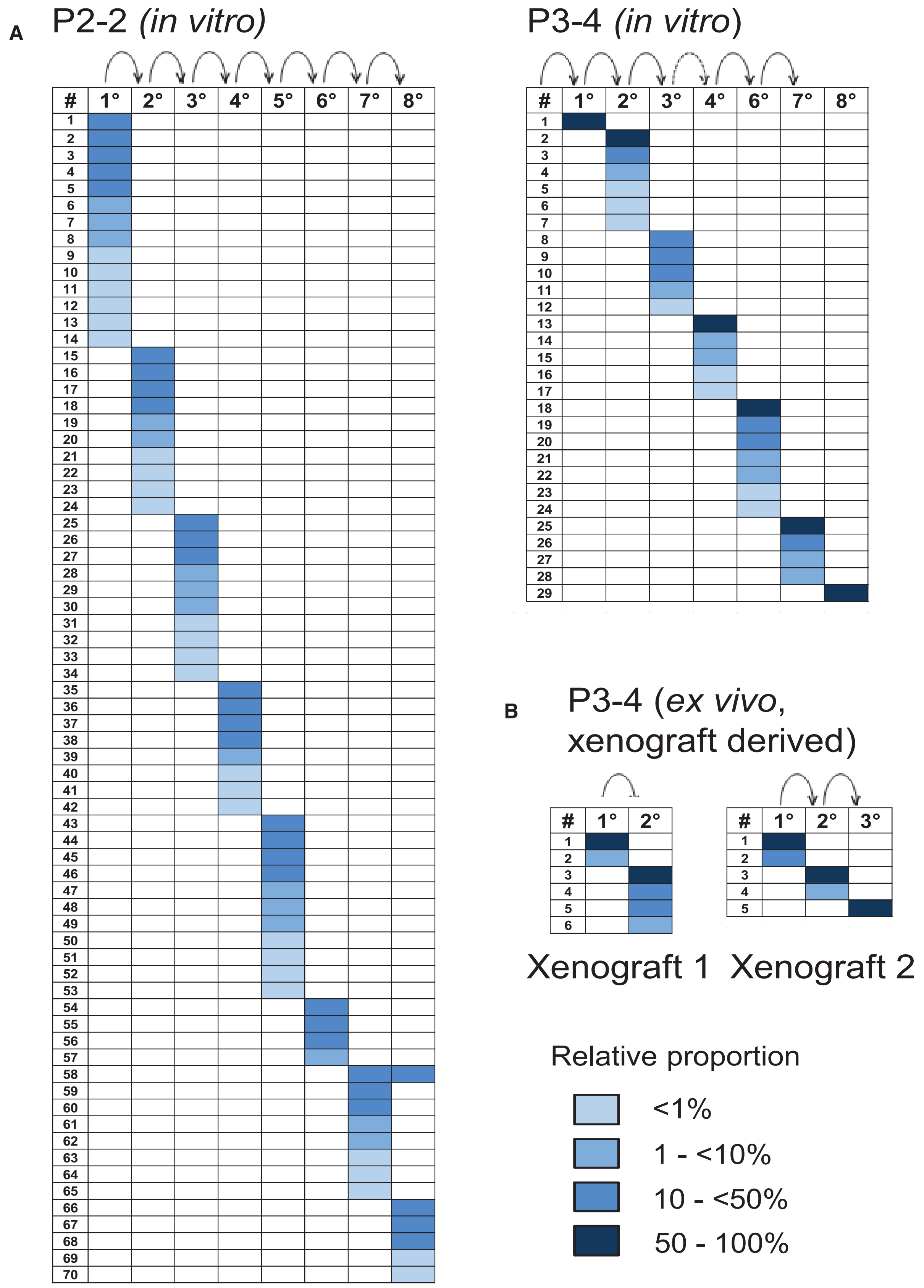

Figure EV2.

© 2017 The Authors
